# Feasibility of Intraocular Projection for Treatment of Intractable Corneal Opacity

**DOI:** 10.1097/ICO.0000000000001852

**Published:** 2019-01-16

**Authors:** Sarah Y. Shim, Songbin Gong, Mark I. Rosenblatt, Daniel Palanker, Ahmed Al-Qahtani, Michael G. Sun, Qiang Zhou, Levi Kanu, Felix Chau, Charles Q. Yu

**Affiliations:** * Department of Electrical and Computer Engineering, The University of Illinois Urbana Champaign, Champaign, IL;; † Department of Ophthalmology and Visual Sciences, The University of Illinois Chicago, Chicago, IL; and; ‡ Department of Ophthalmology, Byers Eye Institute, Stanford University, Palo Alto, CA.

**Keywords:** corneal transplantation, keratoprosthesis, retinal prosthesis, optics, microdisplay

## Abstract

Despite many decades of research and development, corneal opacity remains a leading cause of reversible blindness worldwide. Corneal transplantation and keratoprosthesis can restore corneal clarity, but both have well-known limitations. High-resolution electronic microdisplays may offer an alternative to traditional methods of treating corneal disease using an intraocular implant to project imagery onto the retina, obviating the need for a clear cornea. In this study, we review previous work and recent technologic developments relevant to the development of such an intraocular projection system.

Disease or injury of the cornea may lead to its opacification or scarring. Corneal transplantation can be successful in many cases. However, shortage of donor corneas remains a global problem, with up to 50% of the world population having no access to corneas and ∼13 million on waitlists.^1^ To address this issue, many efforts have been made in the past decades to produce an artificial cornea. Unfortunately, despite progress in this field, no clinically successful biologic artificial cornea is yet available to replace corneas from donors.

In more complicated cases of chronic corneal disease or injury, or after graft failure, vascularization of the cornea may occur, which greatly increases the risk of transplant rejection due to exposure of the graft to the host's immune system.^2^ Keratoprostheses, usually made of nonbiologic transparent materials that are implanted transcorneally, such as the Boston Keratoprosthesis Type I, were developed to address this need.^3^ However, such a design leaves a material/corneal tissue interface that is exposed to the external environment, resulting in a chronic risk of infection. In addition, the presence of a large foreign object in the cornea leads to chronic inflammation, hypothesized to be the cause of a high rate of complications, such as retroprosthetic membranes and glaucomatous optic neuropathy.^4^

Furthermore, currently there is no reliable treatment for eyes suffering from severe ocular surface damage, such as that resulting from thermal or chemical burns. In such cases, the ocular surface lacks the necessary cells to produce mucous and sustain tear films, so that even normal surface exposure leads to persistent epithelial defects and risk of infection. In some cases of severe damage, symblephara may completely cover the ocular surface. The Boston keratoprosthesis type II, which connects the interior of the eye to the surface of the eyelid, can be considered, but it is also associated with a high rate of complications.^5^ Exposure accidents (∼20,000 cases a year), inflammatory conditions of the ocular surface, such as Stevens Johnson syndrome (∼2000 cases a year), mucous membrane pemphigoid (∼500 cases a year), and graft versus host disease (∼5500 cases a year) can result in such severe outcomes.^6–8^ However, despite the surface damage, most of these patients retain an intact intraocular visual pathway.^9^

## RETINAL PROSTHETICS

Retinal prostheses electrically stimulate the inner retinal neurons to restore sight in patients with outer retinal degeneration, such as retinitis pigmentosa or geographic atrophy. Subretinal implants, such as Alpha IMS/AMS (Retina Implant AG, Aachen, Germany)^10,11^ or photovoltaic arrays,^12^ target the bipolar cells and rely on their transmission of the signals to ganglion cells through the retinal network. Epiretinal implants, such as Argus II (Second Sight, Sylmar, CA), stimulate the ganglion cells directly.^13^ Because both types of prostheses bypass part of the retinal signal processing, prosthetic visual code is unlikely to replicate natural vision. The Alpha IMS/AMS and subretinal photovoltaic arrays have light sensors in each pixel and therefore they rely on a clear optical pathway. The Argus system transmits visual information from an external camera to the retinal implant through radiofrequency coils, and therefore, it does not rely on an intact optical pathway. So far, most of the patients with Alpha AMS and Argus II achieved visual acuity below 20/1200, and 2 patients with Alpha IMS demonstrated 20/550 vision.^14,15^

## INTRAOCULAR PROJECTION AS AN ALTERNATIVE TO CORNEAL TRANSPLANTATION OR PROSTHESIS

**FIGURE 1.**
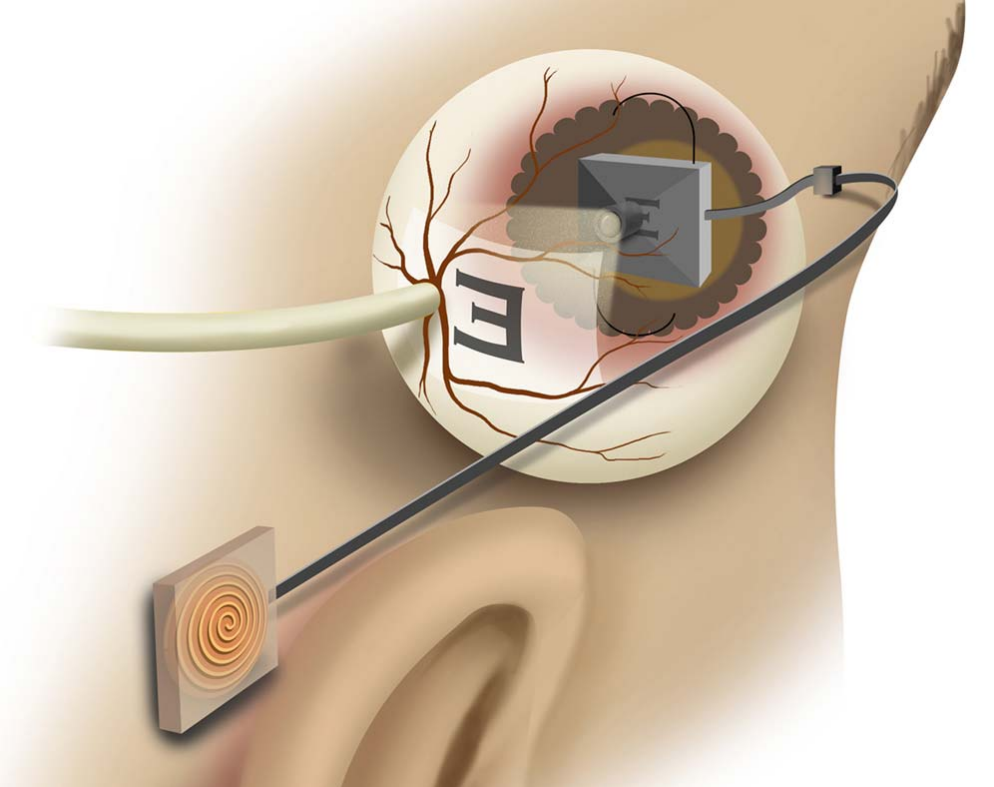
Intraocular projector. A unit implanted behind the ear receives power and video stream data from a radiofrequency transmitter. It delivers power and data to a projector implanted within the eye through a subcutaneous cable. Images on the display are then projected onto the retina, thereby bypassing the opacity of the cornea.

In principle, the Argus system could be used to restore low vision in patients with corneal opacity by electrical stimulation of ganglion cells. However, much better vision might be achieved if the intact retina in such patients could receive optical images from an intraocular electronic display (Fig. 1). By bypassing the anterior visual pathway, such a system would allow patients to see even with opaque corneas or after having lids closed by tarsorrhaphy, as is sometimes required in the treatment of severe ocular surface disease. Such a system would also not have exposed hardware, as with keratoprostheses, or require corneal donors, as with transplantation. Retinal prostheses have already demonstrated the eye's ability to tolerate similar intraocular hardware.^10^

At least 2 patent disclosures discuss such a device.^16,17^ Samiy et al (expired 2009) describe a projection element, with a miniature electronic display, that is coupled with a focusing element. Both are surgically placed in the anterior portion of the eye. Aharoni et al (expires 2022) describe a similar system, all sealed within a large intraocular lens. In the early 2000s, several technologies termed “intraocular vision aid” were developed and described: camera and image processing software,^18^ low-resolution light-emitting diode (LED) display^19^ with an optical system,^20^ and a wireless power and data transmission system.^21^ However, because of technological limitations at the time, primarily with display resolution, no complete functional system was constructed. As a proof of concept and safety test, implants with a single wirelessly powered LED were placed successfully into the anterior segments of 13 rabbits for up to 21 months.^22^ No further work since 2005 was found on this subject.

**FIGURE 2.**
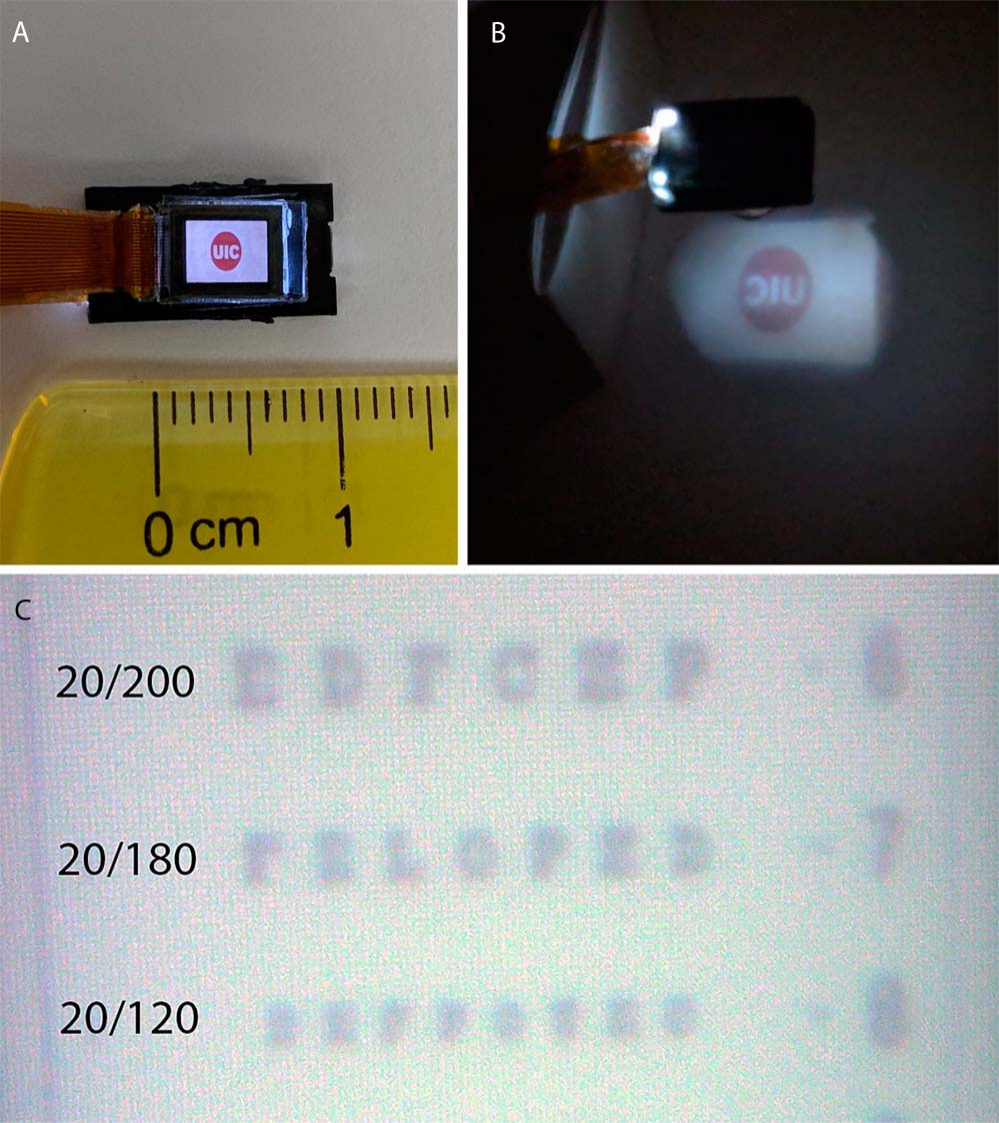
A, Commercially available microdisplay (Kopin) of size 7 × 4 mm, with a pixel pitch of 11 μm, and total resolution of 640 × 480 pixels. B, Image projected from a microdisplay using a projector of a size that can fit within the eye. C, The calculated visual acuity of this system is ∼20/130, which is demonstrated here by projection of an eye chart onto a camera sensor. Contrast can be improved with an optimized lens.

Recent developments of small microdisplays with sufficiently high resolution now enable construction of an intraocular projector. Such displays, used in compact electronic viewfinders and near-the-eye displays, consist of either an LED light source with a liquid crystal display (LCD) or an organic LED array, or an array of micromirrors (liquid crystal on silicone).^23–25^ For intraocular use, LED-backlit LCD has an advantage of a longer lifespan, whereas organic LED provides higher contrast, low power use, and does not require a backlight. Liquid crystal on silicone displays can achieve very high pixel density, but their reflective design makes compact packaging difficult. We have used the Kopin VGA LCD display and a single 3-mm diameter lens to construct a demonstration prototype (Fig. 2).

**FIGURE 3.**
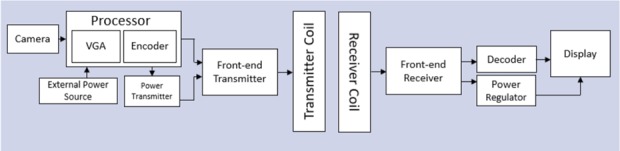
Schematic of a display-based visual prosthesis system. In the external portion, video captured by the camera is encoded by a processor. This signal, along with power, is sent through a radiofrequency transmission coil. In the implanted portion, a receiver unit separates the power and data signals and decodes the visual information to operate the microdisplay.

**FIGURE 4.**
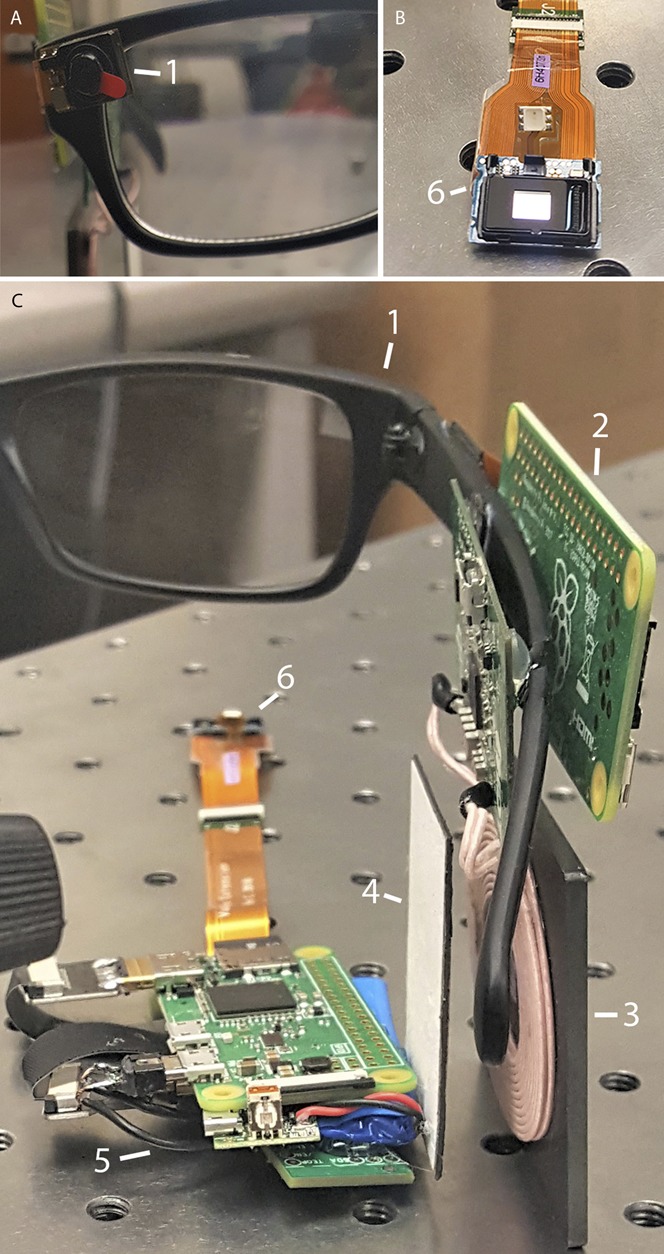
Prototype of the intraocular display electronics. A, External camera (1). B, Microdisplay (6). C, A camera (1) captures video, which is encoded by a processor (2), which sends the data and power through a transmission coil (3) to a receiver coil (4). The signal is then decoded by another processor (5), and the recovered video is displayed by the microdisplay (6).

A complete system for projection of images onto the retina can consist of the following components: first an external camera captures the video imagery. These data are encoded and transmitted wirelessly through the skin, along with the power using radiofrequency coils. A receiver coil and processor implanted outside the eye (behind the ear in the figure) receives the power and visual data and sends it to an intraocular projector through a small transscleral cable. Images on the implanted display are projected onto the retina by a focusing lens (Figs. 1, 3, 4). The intraocular microprojector is best placed close to the anterior segment to provide sufficient distance for image projection onto the retina and away from the sensitive structures of the posterior segment.

## INTRAOCULAR PROJECTION OPTICS

The visual acuity provided by a microdisplay can be calculated from the pixel pitch of the display and the magnification of the optical system used to focus the image onto the retina. A visual acuity of 20/20 corresponds to a pixel pitch of 5 μm on the retina.^26^ The Kopin microdisplay with 11-μm pixel pitch can be projected onto the retina with a magnification ×3 using a single lens, 3 mm in diameter with 3-mm focal length, placed at the object distance of 4 mm and image distance of 12 mm (Fig. 2B). This translates to a 33-μm pixel pitch on the retina, corresponding to a visual acuity of 20/132. Some quality is lost because of optical aberrations, but this still provides better than legally blind vision. We confirm this by projection of our prototype onto a CCD (Fig. 2C).

The 7 × 4 mm display with ×3 magnification mimics viewing the world through a flat display measuring 1.24 × 0.71 m in size, located 1 m away from a standard-sized eye, corresponding to a horizontal visual field of 63.4 degrees. By varying the size of the projected image, different optical focusing designs can have higher visual acuity at the cost of a smaller visual field. Digital magnification of the camera image can also achieve high equivalent visual acuity at the cost of a smaller visual field. Because of the short separation of the projector from the retina, the depth of focus of the optical system is 0.066 mm, which necessitates careful positioning of the implant to ensure a sharp focus on the fovea. Inclusion of an externally adjustable focusing mechanism can potentially reduce the implantation precision needed. To prevent any phototoxicity and discomfort from a projector positioned within the eye, the brightness and the spectral width of the light source can be filtered to be within a physiologic range—on the order of nanowatts per mm^2^.^27^ LED microdisplays are designed for long-term human visual exposure and do not produce ultraviolet or other toxic wavelength light.

## SIZE LIMITS

The size and mass of a device that can be maintained in the anterior segment is limited by the geometry and strength of the supporting tissue. The telescope implanted into the anterior segment in patients with macular degeneration weighs 120 mg.^28^ To reduce the risk of chafing on the interior eye surface, an intraocular projector implanted into the anterior segment should not exceed the size of the crystalline lens, which is about 10 mm in diameter, 6 mm in thickness, and weighs 270 to 300 mg.^29^ Microdisplays measure about 7 mm in length, 1.5 mm in thickness, and weigh in the range of 150 to 200 mg (Fig. 2).^30^ This and the focusing lens need to be contained within a waterproof housing, which can contain inert gas such as nitrogen to allow the device to be neutrally buoyant in an aqueous medium, reducing stress during eye movements. The hardware needed to receive and process power and data cannot be fitted within the eye using current technology, and thus needs to be connected to the projector using a transscleral cable, as is the case with retinal prosthesis systems.

## HEAT AND POWER

Experience with retinal prostheses provides data on heat limits of intraocular electronics. Opie et al demonstrated 135 mW distributed over an area of 5 × 5 mm in contact with the suprachoroidal space to be the safety limit and recommended no more than 19 mW/mm^2^ for implants in contact with the retina.^31,32^ Clinical devices need to conform to international requirements of no greater than 2°C increase at any point on its surface (ISO norm 14708-1 article 17.2). Lazzi^33^ conducted mathematical heat modeling showing a surface temperature increase of 2°C of a conformally coated 4 × 4 mm midvitreous implant at approximately 35 mW. Intraocular projectors have a higher power budget because they are contained within a larger housing. Our demonstration projector power consumption is adjustable from 40 to 75 mW.

## BIOCOMPATIBILITY AND DURABILITY

Any intraocular electronic device must be sealed in a biocompatible and durable waterproof housing. The interior of the eye is an immune-privileged area known to tolerate a variety of materials in the long term. Silicones, acrylics, and titanium are being used successfully in current intraocular implants.^34^ Because light is emitted through the lens, only a power cable needs to exit the housing of the device, making a projector simpler to package than retinal prostheses that have many stimulatory electrodes. Given that device contents remain secure from corrosion, the lifespan will be limited by its fastest wearing component. Current lifetime of the LED backlights is ∼50,000 hours (11 years at 12 hours a day).^35^ LCD displays have a lifetime several times longer than LED backlights.

## SURGICAL TECHNIQUES

**FIGURE 5.**
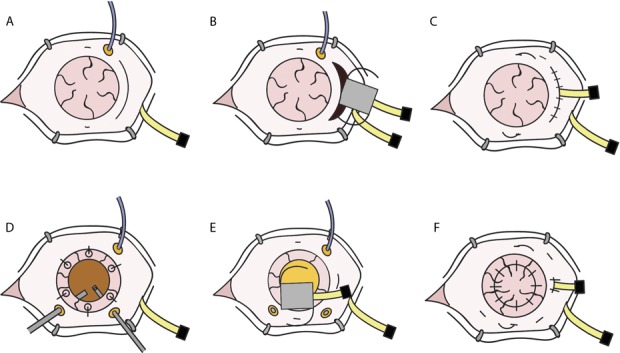
Two methods of implantation. A–C, If the eye has had previous lensectomy and vitrectomy, a pars plana incision can be used. D and E, Alternatively, temporary keratoprosthesis can be used to perform lensectomy and vitrectomy. The projector can be inserted through the trephination incision, and the patient's original corneal button can be sutured back to close the wound. In both cases, the device haptics are externalized through small sclerotomies and fixated to the sclera.

Several approaches could be used for stable implantation of this device in the anterior segment. Implantations into the capsule or ciliary sulcus would be the least invasive and maintain normal anatomic separation of the anterior and posterior segments, but they require an intact capsular bag and appropriately small implants. Fixation to the sclera, either by placing haptics into scleral pockets, melting haptic ends, or suturing to the sclera such as that done for intraocular lenses without capsular support are possible approaches (Fig. 5).^36^ A pars plana incision, scleral tunnel (such as that for extracapsular cataract surgery), or an open sky trephination can be used to insert the device. If the cornea is trephinated once the device is implanted, the patient's own cornea can be placed back, even if opaque (Fig. 5F) The size of surgical wounds could put the patient at risk of expulsive hemorrhage. However, the required incision size can be reduced through incorporation of technologies such as flexible display and housing. The patient's feedback (such as with adjustable sutures for strabismus surgery) or intraocular endoscopy can be used to fine-tune the device's position and focus before conclusion of the surgery.

## SYSTEM LIMITATIONS

Ocular movements normally shift the image on the retina. Because this will not happen with the intraocular projector, as with the Argus retinal implant, patients will have to use head movements and keep their eye steady. Eye tracking can be used to shift the image on the display in accordance with the eye movements, and thereby eliminate this problem.^37^ Medical and surgical complications will likely be similar to those of retinal prostheses and may include leakage of fluid from the eye near the cable crossing the sclera, which may result in hypotony (10% rate in Argus) or infection.^38^ There is also a risk of chronic inflammation if the device is not stably implanted. Prosthetic membranes could grow along the cable or haptics onto the device, although this has not been reported to be common in retinal prostheses. Patients with severe inflammatory eye disease will likely experience an increased rate of complications such as scleral melt as compared with those undergoing retinal prostheses.

The cost of microdisplays is low due to commercial availability (less than $200 per unit), but development, manufacturing, and regulatory expenses will likely result in a first-generation device of similar cost to retinal prostheses ($100,000). This will limit the initial number of patients who are candidates for this device, but with time and refinement, a wider patient pool could benefit.

## CONCLUSIONS

We here review past work and technologies relevant to development of an intraocular projector for treatment of corneal blindness and demonstrate a prototype device. Although much work remains to be performed, including long-term safety and efficacy studies, we believe that an intraocular projection system is within reach of current technology. Such a device will not require donor tissue or corneal implants and has the potential to offer a new paradigm in the treatment of corneal diseases: that corneal clarity is not required for high-quality vision.

## References

[1] Gain P Jullienne R He Z Global survey of corneal transplantation and eye banking. JAMA Ophthalmol. 2016;134:167–173. 26633035 10.1001/jamaophthalmol.2015.4776

[2] Bartels MC Doxiadis II Colen TP Long-term outcome in high-risk corneal transplantation and the influence of HLA-A and HLA-B matching. Cornea. 2003;22:552–556. 12883350 10.1097/00003226-200308000-00013

[3] Saeed HN Shanbhag S Chodosh J The Boston keratoprosthesis. Curr Opin Ophthalmol. 2017;28:390–396. 28383302 10.1097/ICU.0000000000000373

[4] Lee WB Shtein RM Kaufman SC Boston keratoprosthesis: outcomes and complications: a report by the American Academy of Ophthalmology. Ophthalmology. 2015;122:1504–1511. 25934510 10.1016/j.ophtha.2015.03.025

[5] Iyer G Srinivasan B Agarwal S Boston type 2 keratoprosthesis-mid term outcomes from a tertiary eye care centre in India. Ocul Surf. 2018 (ePub ahead of print). 10.1016/j.jtos.2018.08.003 30157458

[6] Islam SS Doyle EJ Velilla A Epidemiology of compensable work-related ocular injuries and illnesses: incidence and risk factors. J Occup Environ Med. 2000;42:575–581. 10874649 10.1097/00043764-200006000-00004

[7] Kohanim S Palioura S Saeed HN Acute and chronic ophthalmic involvement in Stevens-Johnson syndrome/toxic epidermal necrolysis—a comprehensive review and guide to therapy. II: ophthalmic disease. Ocul Surf. 2016;14:168–188. 26882981 10.1016/j.jtos.2016.02.001

[8] Higgins GT Allan RB Hall R Development of ocular disease in patients with mucous membrane pemphigoid involving the oral mucosa. Br J Ophthalmol. 2006;90:964–967. 16613917 10.1136/bjo.2006.092528 PMC1857184

[9] Tsai JH Derby E Holland EJ Incidence and prevalence of glaucoma in severe ocular surface disease. Cornea. 2006;25:530–532. 16783140 10.1097/01.ico.0000220776.93852.d9

[10] da Cruz L Dorn JD Humayun MS Five-year safety and performance results from the Argus II retinal prosthesis system clinical trial. Ophthalmology. 2016;123:2248–2254. 27453256 10.1016/j.ophtha.2016.06.049 PMC5035591

[11] Stingl K Schippert R Bartz-Schmidt KU Interim results of a multicenter trial with the new electronic subretinal implant Alpha AMS in 15 patients blind from inherited retinal degenerations. Front Neurosci. 2017;11:445. 28878616 10.3389/fnins.2017.00445 PMC5572402

[12] Lorach H Goetz G Smith R Photovoltaic restoration of sight with high visual acuity. Nat Med. 2015;21:476–482. 25915832 10.1038/nm.3851 PMC4601644

[13] Luo YH da Cruz L The Argus((R)) II retinal prosthesis system. Prog Retin Eye Res. 2016;50:89–107. 26404104 10.1016/j.preteyeres.2015.09.003

[14] Stronks HC Dagnelie G The functional performance of the Argus II retinal prosthesis. Expert Rev Med Devices. 2014;11:23–30. 24308734 10.1586/17434440.2014.862494 PMC3926652

[15] Stingl K Bartz-Schmidt KU Besch D Subretinal visual implant Alpha IMS—clinical trial interim report. Vis Res. 2015;111:149–160. 25812924 10.1016/j.visres.2015.03.001

[16] Aharoni E Gross Y Intraocular implants. USPTO; 2004. US patent 7 776 087 B2, December 17, 2002.

[17] Samiy N Gerber JDT Systems and methods for projecting an image onto the retina. USPTO; 1994. US patent 5 653 751 A, December 7, 1994.

[18] Krisch I Hijazi N Hosticka BJ Image acquisition and image processing for the intraocular vision aid. Biomed Tech (Berl). 2002;47(suppl 1 pt 1):171–173. 12451806 10.1515/bmte.2002.47.s1a.171

[19] Puettjer D Praemassing F Buss R LED-display for an intraocular microoptic system. Biomed Tech (Berl). 2002;47(suppl 1 pt 1):164–166. 10.1515/bmte.2002.47.s1a.164 12451804

[20] Stork W Eix I Micro displays as intraocular vision aid—design of an optical system. Biomed Tech (Berl). 2002;47(suppl 1 pt 1):161–163. 12451803 10.1515/bmte.2002.47.s1a.161

[21] Hijazi N Krisch I Hosticka BJ Wireless power and data transmission system for a micro implantable intraocular vision aid. Biomed Tech (Berl). 2002;47(suppl 1 pt 1):174–175. 12451807 10.1515/bmte.2002.47.s1a.174

[22] Szurman P Warga M Roters S Experimental implantation and long-term testing of an intraocular vision aid in rabbits. Arch Ophthalmol. 2005;123:964–969. 16009839 10.1001/archopht.123.7.964

[23] Asaki R Yokoyama S Kitagawa H A 0.23‐in. high‐resolution OLED microdisplay for wearable displays. SID Int Symp Dig Tech Pap. 2014;45:219–222.

[24] Richter B Wartenberg P Vogel U Microdisplays for smart eyewear. Optik Photonik. 2018;13:44–47.

[25] Bleha WP Lei LA Advances in Liquid Crystal on Silicon (LCOS) spatial light modulator technology. Proceedings of SPIE. 2013;8736:8.

[26] Dubra A Sulai Y Norris JL Noninvasive imaging of the human rod photoreceptor mosaic using a confocal adaptive optics scanning ophthalmoscope. Biomed Opt Express. 2011;2:1864–1876. 21750765 10.1364/BOE.2.001864 PMC3130574

[27] Lorach H Wang J Lee DY Retinal safety of near infrared radiation in photovoltaic restoration of sight. Biomed Opt Express. 2016;7:13–21. 26819813 10.1364/BOE.7.000013 PMC4722897

[28] Colby KA Chang DF Stulting RD Surgical placement of an optical prosthetic device for end-stage macular degeneration: the implantable miniature telescope. Arch Ophthalmol. 2007;125:1118–1121. 17698761 10.1001/archopht.125.8.1118

[29] Spencer RP Change in weight of the human lens with age. Ann Ophthalmol. 1976;8:440–441. 1267315

[30] Kopin CyberDisplay Specifications. Available at: http://s1.q4cdn.com/801585987/files/doc_downloads/offerings/VGALVSFeatureSheet.pdf. Accessed August 28, 2018.

[31] Opie N Burkitt A Meffin H Heating of the eye by a retinal prosthesis: modeling, cadaver and in vivo study. IEEE Trans Biomed Eng. 2011;59:339–345. 22010144 10.1109/TBME.2011.2171961

[32] Opie NL Greferath U Vessey KA Retinal prosthesis safety: alterations in microglia morphology due to thermal damage and retinal implant contact. Invest Ophthalmol Vis Sci. 2012;53:7802–7812. 23111605 10.1167/iovs.12-10600

[33] Lazzi G Thermal effects of bioimplants. IEEE Eng Med Biol Mag. 2005;24:75–81. 16248120 10.1109/memb.2005.1511503

[34] Chen Y Kim YS Tillman BW Advances in materials for recent low-profile implantable bioelectronics. Materials (Basel). 2018;11:E522. 29596359 10.3390/ma11040522 PMC5951368

[35] Narendran N Gu Y Freyssinier J Solid-state lighting: failure analysis of white LEDs. J Cryst Growth. 2004;268:449–456.

[36] Yamane S Inoue M Arakawa A Sutureless 27-gauge needle-guided intrascleral intraocular lens implantation with lamellar scleral dissection. Ophthalmology. 2014;121:61–66. 24148655 10.1016/j.ophtha.2013.08.043

[37] Caspi A Roy A Wuyyuru V Eye movement control in the Argus II retinal-prosthesis enables reduced head movement and better localization precision. Invest Ophthalmol Vis Sci. 2018;59:792–802. 29392324 10.1167/iovs.17-22377

[38] Humayun MS Dorn JD da Cruz L Interim results from the international trial of Second Sight's visual prosthesis. Ophthalmology. 2012;119:779–788. 22244176 10.1016/j.ophtha.2011.09.028 PMC3319859

